# COVID-19 pandemic: Evaluating its psychological impact and individuals' depression, anxiety, and stress levels

**DOI:** 10.3934/publichealth.2023036

**Published:** 2023-06-13

**Authors:** Bahar Kefeli Çol, Ayşe Gümüşler Başaran, Hilal Pekmezci

**Affiliations:** 1 Guneysu Vocational School of Physical Theraphy and Rehabilitation, Recep Tayyip Erdoğan Üniversity, Rize, Turkey; 2 Faculty of Health Sciences, Recep Tayyip Erdoğan Üniversity, Rize, Turkey; 3 Vocational School of Health Services, Recep Tayyip Erdoğan Üniversity, Rize, Turkey

**Keywords:** coronavirus, mental health, psychological impact

## Abstract

**Background:**

The COVID-19 pandemic is a global public health problem affecting mental health, and basic data are required for evidence-based mental health interventions. This study aimed to identify the prevalence of psychological impacts, anxiety, depression, stress, and any associated risk factors in individuals living in Turkey during the COVID-19 pandemic.

**Materials and methods:**

The population of this descriptive study consisted of individuals over the age of 18 living in Turkey. The data were collected between July–September 2020 using the snowball sampling method. The study was completed with 1733 participants. The data were collected using the Impact of Events Scale-Revised and the Depression Anxiety Stress Scale. Statistical analyses included percentage, mean, standard deviation, a Chi-square test, a Mann Whitney U test, Kruskal Wallis and Tamhane's T2 post hoc, and Spearman's correlation. P < 0.05 was considered statistically significant.

**Results:**

45.1% had moderate or severe psychological effects; 42.7% had a moderate or severe depression, 31.7% had moderate or severe anxiety, and 28.5% had moderate or severe stress levels. Being a woman, being single, unemployment, smoking, the presence of chronic diseases, being young (<35), being a university graduate, having a household size of 5 or more, a low income, having poor health, and being underweight were significantly correlated with some psychological impact, depression, anxiety, and stress levels of people.

**Conclusion:**

During the pandemic period, almost half of the respondents were found to experience some psychological impact of the pandemic and have negative moderate to severe mental health levels. Risk groups for mental health were identified.

## 1. Introduction

The World Health Organization (WHO) declared COVID-19 disease a pandemic, which appeared in China in December 2019, owing to its fast diffusion to Europe and America [1]. In most countries, the government and health institutions have decided to apply some restrictions to reduce the spread rate of the pandemic, to decrease the morbidity and mortality rates, and prevent the pandemic from creating a burden on the health system. Among the protective measures are curfew restrictions, the obligation to use masks, social distancing, travel bans, transition to online education and home office working, temporary closure of workplaces, and quarantine, etc [2]–[4].

As with previous pandemics, the rapid spread of the virus, rapid increases in the morbidity and mortality rates, changes in daily life habits due to the protective measures, loss of income, and fear of losing loved ones during the COVID-19 pandemic negatively affected the mental health of society [5]–[7]. Various studies show that the fear of getting sick, not getting enough health care, dying, staying away from loved ones, stigmatization, discrimination, desperation, and loneliness were risk factors that adversely affected mental health during the pandemic [8],[9]. Recent studies have shown that the frequency of applying to psychiatry emergency service and the risk of suicide increased, especially in people living alone during the quarantine period [10]; additionally, there were great changes in the lifestyle habits of young people who had a more active life before the curfew [11]. It has also been reported that the prevalence of depression and anxiety symptoms increased, the amount of sleep decreased, the quality of sleep and life worsened, and the use of at least one psychotropic drug increased after quarantine [11].

It is a well-established fact that the impact of a pandemic on mental health varies depending on the conditions. Gender, marital status, employment, smoking status, presence of chronic illness, age, education level, size of the household, and personal health perception were reported to be associated with mental health during the pandemic [12]–[19]. For example, women, singles, and those who perceived their health status as poor were reported to be in the higher risk group for mental health problems [20]–[27]. In another study, it was found that women and current and former smokers had worse mental health status compared to men and non-smokers, respectively [11].

It is critical to recognize the COVID-19 pandemic as a serious public health issue since it poses a risk factor for the mental health of the worldwide population. Early diagnosis of mental health problems and determination of high-risk people are necessary for planning evidence-based mental health interventions. Providing mental health protection and necessary health services will reduce the cost of health and increase the quality of service. Therefore, our primary aim in this study was to determine the psychological impact of COVID-19 in society and the prevalence of depression, anxiety, stress, alongside determining the factors affecting mental health.

## 2. Materials and methods

### 2.1. Research population

The research is a descriptive design. The population included approximately 64 million people over the age of 18 living in Turkey. There was no sample selection, and the goal was to reach as many people over the age of 18 who volunteered to participate as possible. The data were collected between July and September 2020 using the snowball sampling method. The snowball sampling method is a non-probability sampling method whose results represent only the participants. The main population of the study consisted of all students and staff of a related university. The main population was then asked to share the survey link with additional people they would recommend. Other participants included those who could be contacted by the main population and other chained individuals who could be reached by them. First, to collect the data, permission was obtained from the related university and a link to the questionnaire created with google forms was sent to the e-mails of students and staff through the Department of Information Technologies. Afterward, the participating students and staff were asked to share the link with other participants using social media tools such as e-mail, WhatsApp, Twitter, Instagram, and Facebook. The inclusion criteria were living in Turkey, being over 18 years of age, and volunteering. Accordingly, the study was completed with 1733 people who agreed to participate in the study and filled out the forms.

### 2.2. Main hypotheses of the research

H_1_: There is a significant difference between the psychological impact and socio-demographic variables.

H_2_: There is a significant difference between the depression level and socio-demographic variables.

H_3_: There is a significant difference between the anxiety level and socio-demographic variables.

H_4_: There is a significant difference between the level of stress and socio-variables.

H_5_: There is a significant positive correlation between the depression level and the psychological impact.

H_6_: There is a significant positive correlation between the anxiety level and the psychological impact.

H_7_: There is a significant positive correlation between the stress level and the psychological impact.

### 2.3. Study tools

The data were collected using a descriptive information form developed by the researchers, the impact of events scale-revised (IES-R), and the depression anxiety stress scale (DASS). The descriptive information form consists of socio-demographic questions investigating gender, age, education level, marital status, number of family members, employment status, monthly income, smoking status, chronic disease history, self-rated health status, and BMI.

#### 2.3.1. The Impact of Events Scale-Revised (IES-R)

The IES-R aims to determine the stress of cases experiencing trauma. On the scale, 22 questions score the severity of symptoms in the last 7 days between 0 and 4. A score of 0 indicates the absence of symptoms and a score of 4 indicates maximum symptoms. The IES-R scale consists of three subscales: intrusion, avoidance, and hyperarousal [28]. The total IES-R scale score was determined as 0–23 (normal psychological effect), 24–32 (mild psychological effect), 33–36 (moderate-psychological effect), and >37 (severe psychological effect). The Turkish validity and reliability of the scale was performed by Çorapçioğlu et al. in 2006 [29]. In this study, the total Cronbach's alpha value of the IES-R was found to be 0.91.

#### 2.3.2. Depression Anxiety Stress Scale (DASS)

**Figure 1.**
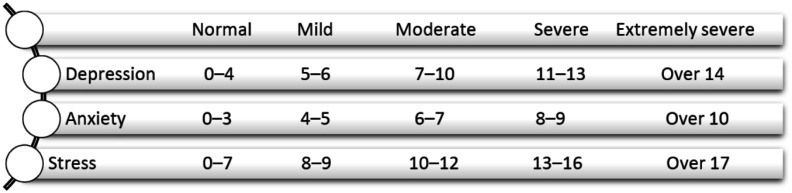
Depression Anxiety Stress levels.

Consisting of 21 items, the DASS-21 is a set of three self-report scales designed to measure the emotional stages of depression, anxiety, and stress [30]. The scale includes 21 questions scoring the severity of depression, stress, and anxiety symptoms between 0 and 3. Depression anxiety stress levels were evaluated according to the score ranges in the figure below (Figure 1). The Turkish validity and reliability of the scale was performed by Saricam in 2018 [31]. In this study, Cronbach's alpha values of the depression, anxiety, and stress sub-scales of the DASS-21 were found to be 0.91, 0.85, and 0.90, respectively.

### 2.4. Statistical analysis

For statistical analysis of the data, the SPSS 22 package program was used. Descriptive data were presented as percentages, mean, and standard deviations. In the analysis of qualitative data, the Chi-square test was used; in the analysis of quantitative data, the Mann Whitney U test, the Kruskal Wallis, and Tamhane's T2 post hoc were used. Spearman's correlation was used to evaluate the relationship between variables. In the correlation analysis, 0–0.19 indicates no correlation, 0.20–0.39 indicates a weak correlation, 0.40–0.69 indicates a moderate correlation, 0.70–0.89 indicates a strong correlation, and 0.90–1.00 indicates a very strong correlation. P < 0.05 was considered statistically significant.

### 2.5. Research ethics

To conduct the research, permission was obtained from the Ethics Committee (40465587–050.01.04–210), and all respondents gave their informed consent prior to their inclusion in the study. Informed consent was obtained from all individual participants included in the study.

## 3. Results

The study showed that 70.9% of the respondents were female, and their average age was 26.3 ± 9.76. Approximately 82.1% were university graduates, 77.8% were single, and 86.6% had a household of 3–4. Approximately 53.5% lived in the city center and 68% were students. Approximately 21.3% smoked, 6.6% used alcohol, 10% had a chronic disease, and 50.7% rated their health as good. The average BMI was 23.93 ± 6.3, and 9.1% were obese.

The total score obtained from the IES-R was 31.23 ± 15.58, which indicated a mild psychological effect, and the DASS-21 mean score was 17.38 ± 13.54. The depression mean score was 6.32 ± 5.33, indicating mild depression, the anxiety mean score was 4.20 ± 4.04, indicating mild anxiety, and the stress mean score was 6.86 ± 5.17, indicating normal levels. The total and subscale scores from both scales are shown in Table 1.

**Table 1.** The Impact of Events Scale (IES-R) and the Depression Anxiety Stress (DASS-21) Scale scores.

The Impact of Events Scale (IES-R)	X	SS	Min–max	(DASS-21) Scale	X	SD	Min–max
Intrusion	10.24	7.04	0–32	Depression	6.32	5.33	0–21
Avoidance	13.02	5.58	0–29	Anxiety	4.20	4.04	0–21
Hyperarousal	7.97	5.49	0–24	Stress	6.86	5.17	0–21
Total IES-R	31.23	15.58	0–81	Total IES-R	17.38	13.53	0–63

In the IES-R, 33.8% of the respondents evaluated the psychological effects of the pandemic as normal, 21.2% as mild, 8.3% as moderate, and 36.8% as severe. When evaluated in terms of depression, 44.1% had normal levels, 11.7% had extremely severe depression, 9.5% had severe depression, 21.5% had moderate depression, and 13.2% had mild depression. In terms of anxiety, 52% had normal anxiety, 10.4% had extremely severe anxiety, 8.8% had severe anxiety, 12.5% had moderate anxiety, and 16.3% had mild anxiety. In terms of stress, 59.7% had normal stress levels, 5.5% had extremely severe stress levels, 9.5% had severe stress levels, 13.5% had moderate stress levels, and 11.8% had mild stress levels.

According to some variables, the analysis of the scores obtained from the IES-R and the DASS-21 scale are shown in Tables 2 and 3. The total median score of the impact of events were found to be significantly higher in those who were female, single, unemployed, smokers, and those experiencing physical symptoms. The examination of the median depression anxiety stress scores showed that gender, marital status, employment status, cigarette consumption, and physical symptoms were significantly different in the three sub-scales, and the presence of chronic disease was found to be significantly different in the anxiety and stress sub-scales.

**Table 2.** Comparison of the scores of the Impact of Events Scale (IES-R) and the Depression Anxiety Stress (DASS-21) Scale with some variables.

Independent variables	N (%)	The Impact of Events (IES-R)	Depression Anxiety Stress (DASS-21) Subscales		
		Total IES-R	Depression	Anxiety	Stress
Woman	1229 (70.9)	916.34	902.73	907.35	922.84
Male	504 (29.1)	746.68	779.87	768.61	730.83
		U = 247209.500 Z = −6.607 P < 0.001	U = 265796.500 Z = −4.656 P < 0.001	U = 260117.000 Z = −5.280 P < 0.001	U = 241077.000 Z = −7.271 P < 0.001
Married	385 (22.2)	769.40	655.24	756.15	709.48
Single	1348 (77.8)	894.87	927.48	898.66	911.99
		U = 221915.000 Z = −4.340 P < 0.001	U = 177961.500 Z = −9.445 P < 0.001	U = 216812.000 Z = −4.964 P < 0.001	U = 198846.500 Z = 7.019 P < 0.001
Employed	415 (23.9)	747.83	713.58	774.19	750.04
Unemployed	1318 (76.1)	904.52	915.31	896.22	903.83
		U = 224029.500 Z = −5.564 P < 0.001	U = 209815.000 Z = −7.185 P < 0.001	U = 234968.000 Z = −4.364 P < 0.001	U = 224945.500 Z = −5.473 P < 0.001
Smoking	369 (21.3)	943.81	952.33	933.72	955.21
No smoking	1364 (78.7)	846.22	843.92	848.95	843.14
		U = 223313.500 Z = −3.324 P = 0.001	U = 220173.000 Z = −3.704 P < 0.001	U = 227038.500 Z = −2.908 P = 0.004	U = 219109.000 Z = −3.826 P < 0.001
Chronic disease	173 (10.0)	934.21	902.84	1007.80	964.25
No chronic disease	1560 (90.0)	859.55	863.03	851.39	856.21
		U = 123312.000 Z = −1.862 P = 0.063	U = 128740.500 Z = −0.996 P = 0.319	U = 110582.000 Z = −3.929 P < 0.001	U = 118115.000 Z = −2.701 P = 0.007
No physical symptoms	1030 (59.4)	773.29	766.20	745.38	760.01
Physical symptoms	703 (40.6)	1004.30	1014.69	1045.18	1023.76
		U = 265523.500 Z = −9.438 P < 0.001	U = 258217.000 Z = −10.183 P < 0.001	U = 236781.500 Z = −12.335 P < 0.001	U = 251841.500 Z = −10.799 P < 0.001

In the Kruskal Wallis analysis performed with variables with three or more groups, a significant difference was found between age, educational status, household size, occupation, monthly income, self-rated health status, BMI, and IES-R scales. In the post hoc analysis, a significant difference was found between the ages of 18–34 and 35–64 (p < 0.001), university graduates and the other two groups (p = 0.001, p < 0.001), those with a household of 5 or more and the other groups (p < 0.001, p = 0.002, p = 0.002), students and public/private sector employees and the other groups (p < 0.001, p < 0.001, p = 0.019), those below the minimum wage and those from the minimum wage to 10,000 (p < 0.001), all groups in their self-rated health status (p < 0.001), and those who were underweight and those who were normal and overweight (p = 0.027, p = 0.001) (Table 3).

**Table 3.** Comparison of the mean scores of the IES-R and DASS-21 Scales Sub-Scales with some variables.

Independent variables		N (%)	The Impact of Events (IES-R)	Depression Anxiety Stress (DASS-21) Subscales		
			Total IES-R	Depression	Anxiety	Stress
			Mean rank	Mean rank	Mean rank	Mean rank
Age	18–34	1428	895.09	913.51	897.51	905.40
	35–64	297	724.21	643.22	719.05	679.29
	65+	8	1154.56	873.56	979.63	981.00
			KW X^2^ = 31.335 P < 0.001	KW X^2^ = 72.189 P < 0.001	KW X^2^ = 32.005 P < 0.001	KW X^2^ = 50.848 P < 0.001
Education level	Primary school- High school	153 (8.9)	764.65	746.91	825.65	781.23
	University	1422 (82.1)	893.59	898.75	878.08	889.65
	Master/ Ph.D.	157 (9.1)	737.93	690.98	801.45	739.93
			KW X^2^ = 23.344 P < 0.001	KW X^2^ = 34.214 P < 0.001	KW X^2^ = 4.503 P = 0.105	KW X^2^ = 17.632 P < 0.001
House hold size	Alone	60 (3.5)	752.13	813.84	761.90	769.79
	2 people	152 (8.8)	862.52	835.41	889.67	823.60
	3–4 people	1500 (86.6)	866.26	866.48	864.21	869.60
	5 people and more	21 (1.2)	1280.40	1284.71	1202.52	1273.05
			KW X^2^ = 1.491 P = 0.001	KW X^2^ = 16.019 P = 0.001	KW X^2^ = 12.627 P = 0.006	KW X^2^ = 17.355 P = 0.001
Monthly income	Minimum wage (2.324)- and below	1056 (60.9)	923.11	940.20	915.55	929.88
	Over minimum wage	642 (37.0)	779.79	755.91	788.87	769.69
	10 thousand and over	35 (2.0)	773.66	696.19	835.44	754.66
			KW X^2^ = 34.009 P < 0.001	KW X^2^ = 58.683 P < 0.001	KW X^2^ = 26.101 P < 0.001	KW X^2^ = 42.916 P < 0.001
Self-rated health status	Poor	40 (2.3)	1494.26	1523.36	1557.36	1535.51
	Moderate	459 (26.5)	1052.12	1060.50	1076.56	1038.97
	Good	983 (56.7)	813.24	815.76	810.88	828.33
	Very good	251 (14.5)	639.05	609.21	593.56	597.41
			KW X^2^ = 189.162 P < 0.001	KW X^2^ = 215.732 P < 0.001	KW X^2^ = 247.473 P < 0.001	KW X^2^ = 205.264 P < 0.001
BMI	Underweight	125 (7.2)	1005.51	989.36	982.39	998.98
	Normal	1033 (59.6)	872.87	883.59	872.39	878.82
	Overweight	418 (24.1)	810.68	816.02	817.63	814.19
	Obese	157 (9.1)	868.02	796.17	871.13	824.71
			KW X^2^ = 15.019 P = 0.002	KW X^2^ = 16.196 P = 0.001	KW X^2^=11.003 P = 0.012	KW X^2^ = 15.118 P = 0.002

In the Kruskal Wallis analysis performed with variables with three and more groups, age, education level, household size, place of residence, monthly income, self-rated health status, and BMI were significantly different in terms of depression. Age, household size, monthly income, self-rated health status, and BMI caused a significant difference in terms of anxiety, and age, educational status, household size, monthly income, self-rated health, and BMI made a significant difference in terms of stress (Table 3). In the post hoc analysis, a significant difference was found in the depression sub-scale in the following items: between the ages of 18–34 and 35–64 (p < 0.001), between university graduates and the other two groups (p = 0.001, p < 0.001), those with a household of 5 or more and other groups (p = 0.003, p = 0.003, p = 0.006), between those living in villages and those living in provinces and districts (p = 0.027, p = 0.039), between students and public/private sector employees, housewives, and retired people (p < 0.001, p < 0.001, p < 0.001, p = 0.003), between those whose monthly income was at or below the minimum wage and the other two groups (p < 0.001, p = 0.010), among all groups in terms of their self-rated health status (p < 0.001), between those who were overweight and obese (p = 0.004, p = 0.011), and between normal and overweight (p = 0.027). In the anxiety subscale, a significant difference was found between those between the ages of 18–34 and 35–64 (p < 0.001), between those with a household of 5 or more and those living alone or with 3–4 people (p = 0.021, p = 0.039), between students and public/private sector employees (p < 0.001, p = 0.003), between those whose monthly income below the minimum wage and those at from the minimum wage to 10,000 or greater (p < 0.001), among all groups in terms of their self-rated health status (p < 0.001), and between those underweight and overweight (p = 0.009). In the stress subscale, a significant difference was seen between those aged 18–34 and 35–64 (p < 0.001), between the university graduates and the other two groups (p = 0.018, p < 0.001), between those with a household of 5 or more and the other groups (p = 0.002, p = 0.003, p = 0.007), between students and public/private sector employees and other groups (p < 0.001, p < 0.001, p < 0.016), between those whose monthly income is at or below the minimum wage and from the minimum wage to 10,000 (p < 0.001), among all groups in terms of their self-rated health status (p < 0.001), and between those who were underweight and overweight and obese (p = 0.001, p = 0.014) (Table 3).

Correlation coefficients between the IES-R and the depression score were calculated as rs = 0.690, indicating a moderately significant positive relationship; the correlation value between the anxiety and stress scores was calculated as rs = 0.718, rs = 0.755, and a strong positive correlation was seen (p < 0.001) (Table 4).

**Table 4.** Correlation coefficients between IES-R Scale and DASS-21 Scale and its Sub-Scales (Spearman).

DASS-21	IES-R Total
Depression	0.690**
Anxiety	0.718**
Stress	0.755**

Note: **p < 0.01.

## 4. Discussion

This study aimed to determine the prevalence of psychological effects, anxiety, depression, and stress in individuals during the COVID-19 epidemic. In this study, the IES-R score, which revealed the psychological effects of the respondents, was found to be moderate and severe (45.1%). Various studies have reported moderate or severe psychological effects between 53.8% and 23.6% [19]–[22],[25]–[27]. In this study, the moderate or severe depression, anxiety, and stress rates experienced by the respondents were 42.7%, 31.7%, and 28.5%, respectively. Evidence showed that the rate of moderate 0or severe depression ranged from 37.25% to 16.5%, anxiety from 39.08% to 28.8%, and stress from 34.12% to 8.1% [19]–[21],[25],[27]. The variations in findings might be attributed to variances in the number of patients and the pandemic period in countries where the psychological effects, depression, anxiety, and stress during the Covid-19 pandemic were measured. The findings demonstrated that the pandemic mostly caused major mental problems like depression, anxiety, and stress globally.

In this study, female respondents had significantly higher scores on the IES-R, DASS-21 depression, anxiety, and stress scale, which was consistent with previous studies suggesting that psychological effects [20]–[27], depression, anxiety, and stress were more common in women after traumatic events [21],[25],[26]. Other studies evaluating the impact of Covid-19 on mental health also show that the female gender is a risk factor for poor mental health [12]–[15]. Women's biological structure, physiological reactions, social and cultural position, method of coping with stress, the role of motherhood, the meaning they attribute to the pandemic, and economic factors can have an impact on their mental health [32],[33].

Single respondents in the study scored significantly higher on the IES-R and the DASS-21 depression, anxiety, and stress scale. Being single is emphasized to increase the psychological impact [21],[24], and negatively affect depression, anxiety, and stress levels [15],[21]. Marriage is shown to be a crucial element in protecting and improving psychological health, as well as giving social and psychological support [33],[34].

However, although some research links being single to poor mental health [11],[20], others demonstrate that marital status is either unrelated to mental health [25] or that being married is a risk factor for poor mental health [17]. Since there are inconsistent results in the literature on the effects of marital status on mental health during the pandemic process, it is recommended to evaluate the effects of marital status with larger participants in future studies.

It was determined that unemployed respondents in this study had significantly higher scores on the IES-R, DASS-21 depression, anxiety, and stress scale. Likewise, relevant studies show that unemployed people report higher levels of psychological impact [18],[19], stress, anxiety, and depression [16],[20]. Employment is one of the main determinants of health. Unemployment is a risk factor for mental health, and mental problems are more common in unemployed groups compared to employed groups [35],[36]. The impact of unemployment on impaired mental health can be explained by financial difficulties, loss of social status (loss of social resources/social isolation), uncertainty, and insecurity in finding a job [35],[37],[38].

In the study, smokers scored significantly higher on the IES-R scale. In their investigation of the association between smoking, exposure to traumatic events, and post-traumatic stress, Feldner et al. (2008) found that psychological consequences were more prevalent in smokers following traumatic events [39].

Smokers got significantly higher scores on the DASS-21 depression, anxiety, and stress scale. A study evaluating the effect of Covid-19 on mental health also emphasized that smoking was a risk factor for poor mental health [15]. A relationship between smoking and depression [40], anxiety [41], and stress [42] is highlighted in the literature. Many national and international newspapers, magazines, and media channels around the world reported that smokers were prone to Covid-19 infection due to weakened lung function, cross-infection, and sensitive hygiene habits; additionally, smokers had more adverse disease prognoses, intensive care unit hospitalizations, and mortality [43],[44]. Considering this information, it was expected that the mental health of smokers would be adversely affected during the pandemic.

The presence of chronic disease was not found to be a significant factor in the IES-R scale score. However, relevant studies showed that those with a history of chronic disease experience the psychological effect of the pandemic [20],[22],[25],[26]. The reason for the difference between this finding and other findings may have been because of the low number of respondents with chronic diseases in this study. In further studies the number of respondents with chronic diseases should be explored.

The presence of chronic disease led to a significantly higher score on the DASS-21 anxiety and stress scale, which were consistent with studies suggesting that those with chronic illnesses suffer from higher levels of psychological symptoms [15],[25],[26],[37],[45]. This situation may be explained by identifying chronic illnesses as the leading cause of mortality from Covid-19 and raising the likelihood of getting the condition [44]–[46].

Young respondents (<35) got a significantly higher score on the IES-R scale. Similarly, several studies have found that younger people are more mentally affected [20],[22]–[24]; nevertheless, others have claimed that either older people are more affected [18] or that age has no influence [46].

Young respondents (<35) also received significantly higher scores on the DASS-21 depression, anxiety, and stress scale. There are similar findings in the literature [15],[25]. Although some research examining the pandemic's influence on mental health reveals that young age is a risk factor for poor mental health [12],[14],[17],[26], others do not [20].

It is highlighted in the literature that the rate of use of social media by young people is high and that the rate of internet access decreases as age increases [47]. The young age group may be in the high-risk category because they are overwhelmed with misinformation and rumors whose accuracy and reliability are not confirmed by the social environment and media. The public's perception of the pandemic threat appears to be based on rumors and untrustworthy sources rather than official health authorities [48],[49]. As a result, unconfirmed information and rumors can be risk factors for depression, anxiety, and stress [50],[51]. Due to the inconsistencies in the literature regarding the effect of the pandemic on different age groups, it would be beneficial to consider the use of social media in age groups in further studies.

University graduates got significantly higher scores on the IES-R scale. This result is similar to a study by El-Zoghby et al. (2020) evaluating the impact of the Covid-19 pandemic on mental and social health [22].

University graduates also got significantly higher scores on the DASS-21 depression and stress scale, which was not consistent with other studies of Covid-19 in the literature [21],[25]. This outcome can be explained by the economic downturn triggered by COVID-19, which caused concern regarding employment and elements of the future, as well as financial uncertainty among university graduates.

It was determined in the study that those with a household of 5 or more had a significantly higher score on the IES-R scale. Other relevant studies emphasized that as the number of people in the household increases, the psychological effect increases [20],[24].

This study revealed a significantly higher score on the DASS-21 depression, anxiety, and stress scale for those with a household size of 5 and above. Our study confirmed literature findings, demonstrating that the smaller the population in the house, the better the family's mental health [21]. This result may be due to discussing COVID-19 too much among family members. Besides, every member of the household could worry about other family members in the risk group. As the number of people in the household increased, the level of anxiety increased, and mental health could be adversely affected.

The respondents with a low income had significantly higher scores on the IES-R and DASS-21 depression, anxiety, and stress scale scores. The low-income level is a risk factor for poor mental health [12],[15],[21]. Those without sufficient financial resources may have been forced to cut their spending due to the income shock caused by the Covid-19 health crisis [51]. Therefore, restrictions on basic needs such as housing, clothing, food, education, health, cleaning, and personal care may pose a risk for poor mental health.

Those who rated their health as poor got significantly higher scores on the IES-R and DASS-21 depression, anxiety, and stress scales, which was consistent with the literature, revealing that their self-rated poor health leads to higher levels of psychological effect [32],[48], stress, anxiety, depression [25],[27], and mental health [26],[37]. This may have been because people who perceived their health as poor felt vulnerable and powerless to get sick and recover, and that the effects of Covid-19 on people in poor health were more severe [47].

Our study demonstrated that those with low BMI scored significantly higher on the IES-R, DASS-21 depression, anxiety, and stress scales. Low body mass index is reported to be a risk factor for anxiety and stress levels [48]–[50]. The reason for this may be that underweight people have lower body resistance and, accordingly, their susceptibility to diseases increases. No studies, to our knowledge, evaluated the effect of low body mass index on mental health during the Covid-19 pandemic. Therefore, it is recommended to conduct studies evaluating the effect of low body mass index on mental health and underlying factors during the pandemic.

A strong or moderate positive relation was identified in the correlation study between the scale of the impact of events and the depression, anxiety, and stress scale, indicating that the mental health of people impacted by the pandemic was also negatively affected.

## 5. Conclusions

It is concluded that about half of the participants were psychologically affected by the pandemic at a moderate or severe level. One-fifth of the participants are above moderate, and about half experienced moderate or severe depression, anxiety, and stress. For mental health, female gender, being single, unemployment, smoking, presence of chronic disease, young age, being a university graduate, the size of household, low income, poor health, and poor body mass index (BMI) were found to be risk factors.

For this reason, in extraordinary situations such as pandemics that may be experienced in the next periods, it is necessary to start studies on mental health as soon as possible. It is recommended to intervene primarily in groups (women, smokers, etc.) that are known to be more affected.

Considering the integrity of the health service, public health nurses have significant functions in determining, protecting, developing, and improving the mental health of society. In the planning of the nursing services to be provided, the first step is to define the priority problems and the risk groups in which these problems develop. It is recommended to ensure support and consultancy services for all individuals in society by prioritizing high-risk groups.

## 6. Limitations

The limitations of this study are as follows.

The snowball sampling strategy was not based on a random selection of the sample, the study population did not reflect the real model of the general population. The number of young population in the sample is high and leading to selection bias. As a result, the conclusion was less generalizable to people with less education (e.g., primary school, high school). Finally, our findings cannot be generalized to these groups due to the low number of participants with high income, over 65 years of age, and chronic diseases. Despite the above limitations, this study provides the basis for future research by providing important insights into psychological responses to the normalization process of the COVID-19 pandemic. In addition, it will guide the determination of the priority groups (e.g., Single, Smoking and BMI) to be intervened.

## 7. Implications for nursing practice

In the planning of the nursing services, the first step is to define the priority problems and the risk groups in which these problems develop. Various studies show that the Covid-19 pandemic increases the psychological impact, depression, anxiety, and stress levels of society. Considering the integrity of the health service, nurses have significant functions in determining, protecting, developing, and improving the mental health of society. Education and counseling approaches to be made for the benefit of society, with a priority for high-risk groups, will focus on this goal. In this context, the determination of the content of the training to protect, improve and develop the mental health of society will guide the nurses in the process of developing new strategies and consultancy services.
